# Evaluation of the Effect of Temperature (20–700 °C) on the Properties of Prestressing Steel Using AE Signals and FEM Analysis

**DOI:** 10.3390/ma19010023

**Published:** 2025-12-20

**Authors:** Anna Adamczak-Bugno, Sebastian Michał Lipiec, Jakub Adamczak

**Affiliations:** 1 Faculty of Civil Engineering and Architecture, Kielce University of Technology, Av. 1000-An. of Polish State 7, 25-314 Kielce, Poland; jakubadamczak123@gmail.com; 2 Faculty of Mechatronics and Mechanical Engineering, Kielce University of Technology, Av. 1000-An. of Polish State 7, 25-314 Kielce, Poland; slipiec@tu.kielce.pl

**Keywords:** prestressing steel, uniaxial tensile test, stress and strain curve, acoustic emission method, finite element method

## Abstract

**Highlights:**

**What are the main findings?**

- High temperature causes significant strength degradation and changes the failure mechanism.
- AE parameters (Counts to Peak, RA-value) effectively identify the onset of damage.

**What is the implication of the main finding?**

- AE was correlated with the *ε*_22_ strain and von Mises stress (FEM).
- A criterion was developed to determine the moment of load-bearing capacity loss after fire exposure.

**Abstract:**

The study presents a comprehensive analysis of the effects of high temperatures (500 °C and 700 °C) on the microstructure, mechanical properties, and acoustic emission (AE) parameters of cold-drawn prestressing steel. The investigations included mechanical testing, AE signal acquisition, and numerical verification using the finite element method (FEM). It was demonstrated that increasing temperature leads to significant microstructural changes (pearlite spheroidisation, carbide coarsening), resulting in strength degradation and a shift in the failure mechanism from quasi-brittle (initial state) to transitional (500 °C), and finally to ductile (700 °C). For the first time, AE parameters (Counts to Peak and RA-value) were correlated with local axial strains *ε*_22_ and von Mises equivalent stress, enabling the identification of the moment of onset load-bearing capacity loss and the determination of critical material damage thresholds. A multi-criteria diagnostic indicator was proposed to assess the condition of prestressing steel after fire exposure. The results confirm the high potential of AE as a non-invasive tool for evaluating the safety of prestressing tendons and cables in reinforced concrete structures subjected to overheating or fire.

## 1. Introduction

In recent years, a dynamic development of metallurgical technologies has been observed, which has led to the creation of modern grades of reinforcing steels characterised by increased strength and service durability. Of particular importance within this group of materials are prestressing steels, widely used as reinforcement in prestressed concrete (PC) elements. Owing to a carefully selected chemical composition, including an increased carbon content and controlled alloying additions, as well as the use of advanced thermo-mechanical treatment processes such as strain hardening, quenching, and tempering, it is possible to achieve very high yield strength levels (1600–1800 MPa) [1,2,3]. The use of prestressing steels enables a significant reduction in structural weight, an increase in load-bearing capacity and stiffness, and an improvement in resistance to cracking and creep. As a result, these steels are finding increasingly wide application in bridge and building engineering, as well as in structures with elevated durability requirements [4,5].

Among the factors affecting structural elements, those associated with the influence of high temperatures and fire are particularly dangerous and difficult to predict. Thermal actions lead to complex material degradation processes that can significantly reduce the load-bearing capacity and structural integrity. Current building standards regarding the fire resistance of reinforced and prestressed concrete elements mainly account for the degradation of concrete, while knowledge about the behaviour of prestressing steel during and after fire exposure remains limited. Despite research conducted on the effects of temperature on the mechanical properties of prestressing tendons, there are still difficulties in reliably assessing their residual load-bearing capacity after a fire. Previous studies have focused primarily on conventional reinforcing steels with lower strength (235–520 MPa), whereas data concerning cold-drawn prestressing wires and strands remain fragmentary and incomplete [6,7,8,9].

The specific microstructure of prestressing steels, formed as a result of the cold drawing process, is characterised by a high level of residual stresses and pronounced microstructural anisotropy. These features significantly affect the degradation mechanisms occurring during heating and cooling, leading to microstructural transformations that differ from those observed in conventional reinforcing steels. With increasing temperature, phenomena such as recrystallization, pearlite spheroidisation, and carbide coarsening may be observed, which can result in a change in the failure mode from ductile to brittle or dispersed [10,11]. In engineering practice, the lack of unambiguous criteria for assessing the ability of prestressing steel to carry stresses after thermal exposure often results in overly conservative design decisions. Consequently, entire prestressing cables or tendons are replaced, even when damage has occurred only in a portion of the cross-section. This generates significant costs and delays in repair and rehabilitation processes of structures [12,13,14,15].

In this context, the importance of modern non-destructive diagnostic methods (NDT) is increasing, as they enable fast, precise, and reliable assessment of the material condition after exposure to high temperatures. The application of NDT techniques helps reduce the number of destructive tests and provides information on structural and mechanical changes occurring within the material [16,17,18,19]. One of the most promising methods in this field is Acoustic Emission (AE), which is based on recording elastic waves generated as a result of microplastic processes and the initiation and propagation of microcracks [20,21,22,23,24]. Despite the wide use of AE in the diagnostics of concrete, composites, and metals, its application in the assessment of prestressing steels after fire exposure remains limited. The lack of dedicated interpretative criteria makes it difficult to quantitatively evaluate the degree of material degradation, which constitutes a significant barrier to its practical implementation [25,26,27,28]. Several studies have applied acoustic emission to monitor damage development in concrete and metallic materials [29,30] and AE has also been combined with numerical modelling in broader structural analyses, particularly for the interpretation of cracking and stress redistribution in concrete and steel components [29,31]. However, these approaches have generally focused on global behaviour or on conventional reinforcing steels, and they do not address the local relationship between AE parameters and finite-element stress or strain fields in cold-drawn prestressing steel after exposure to high temperature. Consequently, the correlation between AE activity and local mechanical fields relevant to damage initiation in fire-affected prestressing steel remains insufficiently explored. Recent studies on prestressed concrete structures and fire-performance assessment further emphasise the need for reliable post-fire diagnostic procedures for tendons and prestressing systems [32,33,34,35].

For this reason, the aim of the present study is to conduct a comprehensive analysis of the influence of high temperature (500 °C and 700 °C) on the microstructure, mechanical properties, and acoustic emission (AE) behaviour of cold-drawn prestressing bars. In the context of the performed analyses, a decision criterion was developed to identify the onset of load-bearing capacity loss based on a combined evaluation of two AE parameters (Counts to Peak and RA-value) and their corresponding numerical indicators (axial strain *ε*_22_ and von Mises equivalent stress). This multi-criteria approach enables more precise detection of the transition from elastic–plastic deformation to strain localisation and crack initiation, thereby improving the reliability of diagnostics and prediction of residual load-bearing capacity after thermal exposure.

The novelty of this work lies in the coordinated application of microstructural analysis, mechanical testing, acoustic emission monitoring, and numerical verification (FEM) to evaluate degradation mechanisms in prestressing steel subjected to high temperature. For the first time, AE parameters (Counts to Peak and RA-value) were correlated with local FEM-derived plastic strains *ε*_22_ and von Mises equivalent stresses, allowing unambiguous identification of the transition from a quasi-brittle to a ductile failure mechanism. This integrated methodology links microstructural processes with the macroscopic mechanical response and provides the foundation for a quantitative, AE-based diagnostic criterion for assessing the condition of prestressing elements after fire exposure.

## 2. Materials and Methods

To accomplish the research objectives and verify the influence of high temperature on the microstructure, mechanical properties, and acoustic emission signals of prestressing steel, a comprehensive program was developed that included both experimental investigations and numerical analysis. The program consisted of three main stages:

- Preparation and characterisation of the test material, including analysis of chemical composition and microstructure after different heat treatment conditions;
- Conducting mechanical tests and registering acoustic emission during loading of the specimens to failure;
- Performance of numerical analyses to interpret the behaviour of the material after thermal exposure and to validate the experimental results.

**Figure 1:**
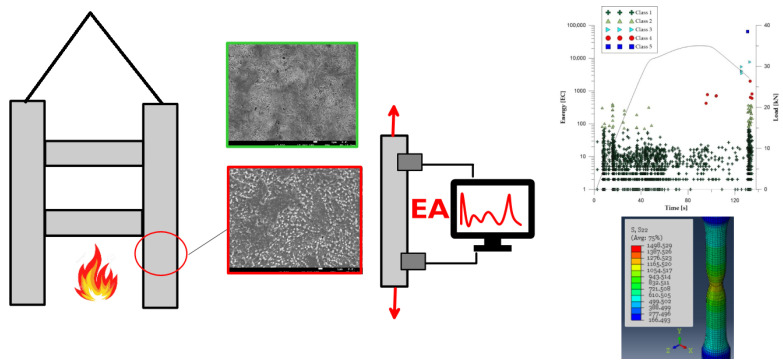
Scheme of testing methods used to assess prestressing steel after exposure to fire temperatures.

The study used a hybrid approach to assess the material after exposure to heat. The assessment considered the results from various stages of the research (see Figure 1). In the first stage, the focus was placed on identifying microstructural changes occurring in prestressing steel under high-temperature conditions corresponding to fire exposure. The second stage involved the assessment of strength properties and the analysis of AE signals during tensile tests, which made it possible to determine the relationships between microstructural parameters, material behaviour, and acoustic emission activity. The third stage, based on numerical analysis, enabled the correlation of the mechanical response of the specimens with the development of damage.

### 2.1. Materials

The material used in the study was a steel grade intended for the production of prestressing bars, containing approximately 0.85% carbon, 0.34% silicon, and 0.42% manganese. The phosphorus and sulfur contents were 0.024% and 0.045%, respectively, while the amounts of chromium, molybdenum, and nickel were low, at 0.048%, 0.001%, and 0.042%. The high carbon content supports its use as a material for prestressing bars in concrete structures, as it has a beneficial effect on the strength and hardenability of the steel.

Within the present work, several treatment variants of the tested material were adopted, which were subjected to both experimental analysis and numerical simulations. The steel in its initial state (denoted as variant ***A***) was used as the reference material. To determine the influence of high temperatures corresponding to fire conditions, the specimens were exposed to controlled thermal treatment. The annealing process was carried out under laboratory conditions, maintaining the temperature for one hour at two levels: 500 °C (variant ***B***) and 700 °C (variant ***C***). In both cases, air was used as the cooling medium.

The exposure time of one hour was selected to ensure that the specimens had sufficient time to reach the target furnace temperature before testing. The heating process followed the operating characteristics of the furnace, without explicit control of the heating rate. After reaching the set temperature, each specimen remained inside the furnace for one hour to stabilise its thermal condition. Once the holding period was completed, the specimens were removed from the furnace and cooled in still air. The same cooling procedure was applied for all temperature variants to maintain consistency across tests.

**Figure 2:**
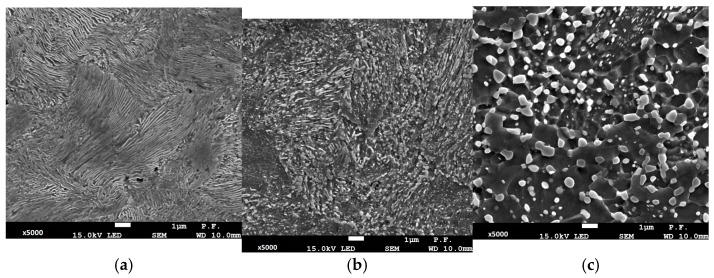
Microstructure of the analysed steel: (**a**) in its initial state (variant ***A***) and after heat treatment: (**b**) variant ***B***, (**c**) variant ***C***.

In the initial state (***A***), the analysed steel was characterised by a microstructure composed of pearlite and proeutectoid cementite, as shown in Figure 2. The observed microstructural features are typical of pearlitic steels (Figure 2a). The applied heat treatment corresponding to fire conditions caused distinct changes in the microstructure of the tested material. The annealing process led to pearlite spheroidisation, growth of carbide particles, and their coagulation. This process intensified with increasing annealing temperature. At 500 °C (variant ***B***), both pearlitic areas and numerous carbide particles are visible in the microstructure (Figure 2b), whereas at 700 °C (variant ***C***), the microstructure is dominated by numerous well-developed carbide particles (Figure 2c).

### 2.2. Methods

To determine the influence of high-temperature exposure on the microstructural, mechanical, and acoustic emission characteristics of prestressing steel, a comprehensive program of experimental investigations and numerical analyses was carried out. It included mechanical testing, registration of acoustic emission phenomena accompanying the deformation and failure processes of the material, and numerical simulations, which enabled a more in-depth interpretation of the observed phenomena and verification of the experimental results.

#### 2.2.1. Uniaxial Tensile Testing

**Figure 3:**
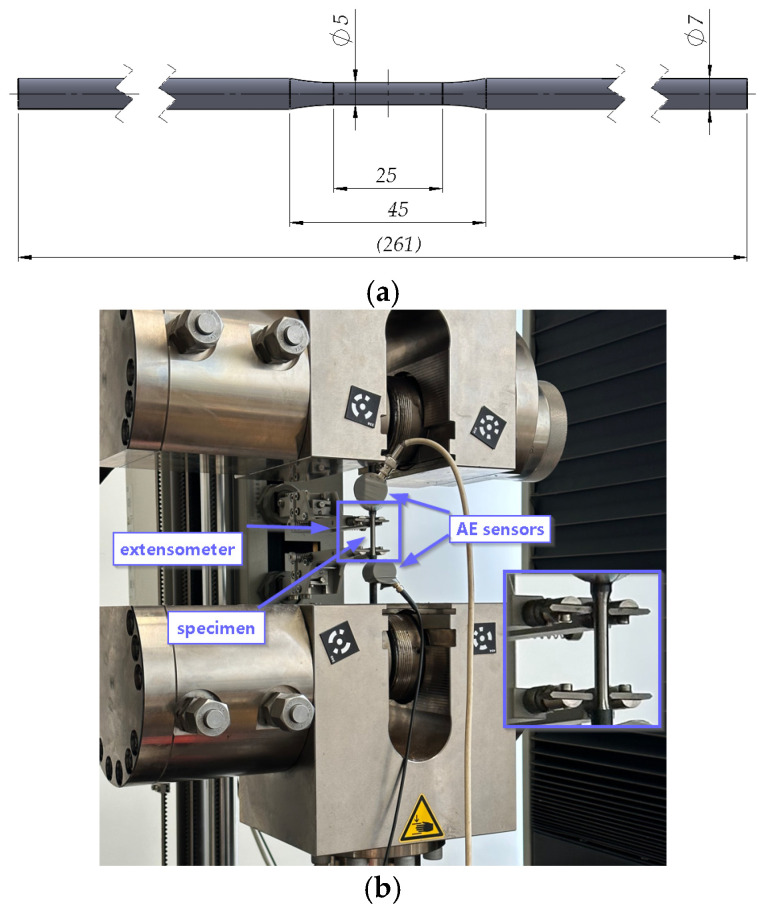
Uniaxial tensile tests: (**a**) dimensions of the specimens accepted for testing, (**b**) view of the specimen mounted on the testing machine with acoustic emission sensors.

To determine the strength and plastic characteristics of the analysed steel after thermal exposure, uniaxial tensile tests were carried out. Specimens in the form of rods with an initial diameter of 5 mm and a measurement base of 25 mm were prepared for testing [36,37,38] (Figure 3a). The laboratory tests were accompanied by an extensometer to measure the elongation of the measuring section of the specimens and acoustic emission sensors (Figure 3b). For each heat-treatment variant, at least two specimens were tested in order to ensure the reliability of the obtained results.

Cold-drawn prestressing steel typically exhibits high microstructural uniformity and low variability in mechanical response. This was confirmed by the small scatter observed between replicates in *σ*_YS_, *σ*_UTS_ and elongation, which supports the adequacy of the adopted specimen size.

The uniaxial tensile tests were conducted under displacement control at a loading rate of 2 mm/min, corresponding to an average strain rate of approximately 1.33 × 10^−3^ s^−1^ for the 25 mm gauge length.

The tests were carried out on a Zwick universal testing machine, which enables precise control of the loading process. During the tests, the duration of the experiment, the applied tensile force, and the displacement measured along the loading axis were recorded. The maximum values of the load applied to the specimens and the displacement are summarised in Table 1. The specimens were loaded until failure, which allowed for a full assessment of their strength properties after exposure to high temperature.

#### 2.2.2. Acoustic Emission Method

In the acoustic emission tests, an AEWin data acquisition system was used, equipped with 8-channel Express-8 cards, enabling simultaneous recording of signals from multiple sensors and their real-time analysis. Signal acquisition was carried out using two Vallen VS75-SIC-40dB sensors with integrated preamplifiers of 40 dB gain, characterised by high sensitivity within the frequency range relevant for the recorded microplasticity phenomena and crack initiation. The sensors were mounted on the specimen surface in its central section by means of a steel clamp, which ensured stable mechanical coupling and minimised the risk of displacement during loading.

Two AE sensors were used to record global acoustic activity during the uniaxial tensile tests. Because the specimens were axisymmetric and the objective was not event localisation, this configuration was sufficient for reliably capturing the overall AE response.

Technical silicone was used as the coupling medium, providing proper acoustic matching between the sensor and the test surface and eliminating the presence of air gaps that could attenuate AE wave propagation. Before each measurement series, a Hsu–Nielsen test (pencil lead break test) was performed to verify correct sensor installation and signal transmission quality.

During the actual tests, acquisition parameters were set to ensure high temporal resolution and minimise background noise: the preamplifier gain was 40 dB, the detection threshold was set to 40 dB, and the sampling frequency was established at 10 MHz.

The parameters chosen for analysis were “Counts to Peak” because it reflects the cumulative AE activity associated with the onset of strain localization, and the “RA value” parameter, commonly used to distinguish between sudden (quasi-brittle) and gradual (ductile) deformation mechanisms. These two parameters therefore provide complementary information, enabling the identification of the transition from uniform plasticity to localised failure.

#### 2.2.3. Numerical Analysis

In order to realise the hybrid approach to analysing the state of the material after exposure to fire temperatures, the results of laboratory tests were supplemented with analyses using AE signal recording and the results of numerical simulations. Comparing and complementing the results from the different types of sources allows a broad analysis of the material’s behaviour under load. Carrying out numerical simulations using FEM allows the determination of individual mechanical quantities that characterise the state of the material. The most common numerically determined quantities taken into account in the strength criteria are stress (components of the stress tensor, effective stress, principal stress, invariant of the stress tensor and stress deviator), strain (individual components of the strain tensor or effective plastic strain), stress triaxiality factor, Lode parameter and others. The axial strain component *ε*_22_ was used because it represents the dominant deformation mode in uniaxial tension and directly reflects strain localisation in the necking region. The von Mises equivalent stress was selected as a scalar measure of the overall stress state, allowing consistent comparison with AE activity and experimental observations. In order to ensure reliable results from numerical simulations, proper preparation of the model must be taken care of. Important steps are the mapping of the geometry of the specimen or component being analysed, the correct definition of boundary conditions, the development of a high-quality finite element (FE) mesh and the correct preparation of the material model. The preparation of the material model, i.e., the relationship between true stresses and strains, requires particular attention and work [39,40,41,42,43]. The programme requires the definition of the linear-elastic and plastic parts of the material. In the research, great attention was paid to the definition of the material for use in numerical simulations, following the methodology presented in the work [41,43,44,45]. As part of the research presented in this paper, non-linear simulations were carried out in Abaqus program.

**Figure 4:**
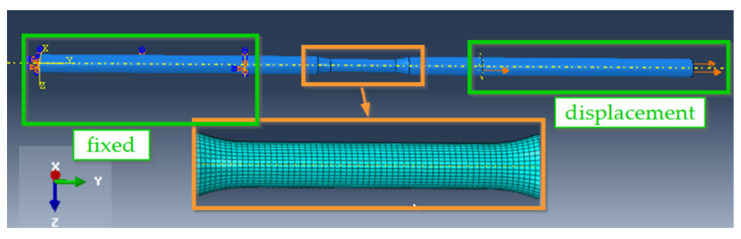
Numerical model of a tensile specimen.

Numerical simulations using FEM were carried out to determine quantities such as stresses and strains that characterise the materials analysed. The models were developed and their loading simulated in Abaqus [46]. The program created numerical models of specimens identical to those used in laboratory tests. The uniaxial tension of the numerical specimens was simulated. Three-dimensional 8-node finite elements of type C3D8R were used in the analysis. The sensitivity of the mesh and its impact on the simulation results were assessed on the basis of preliminary calculations with different finite element (FE) sizes. It was assumed that if the results from the numerical model change only slightly when the FE mesh size is changed, they can be considered objective. In this way, the size of the smallest finite element in the final numerical model was selected as 0.4 mm. The boundary conditions in the numerical model corresponded to a laboratory tensile test: the surface of the specimen was fixed on one side, while a displacement load was applied on the other side (see Figure 4).

**Figure 5:**
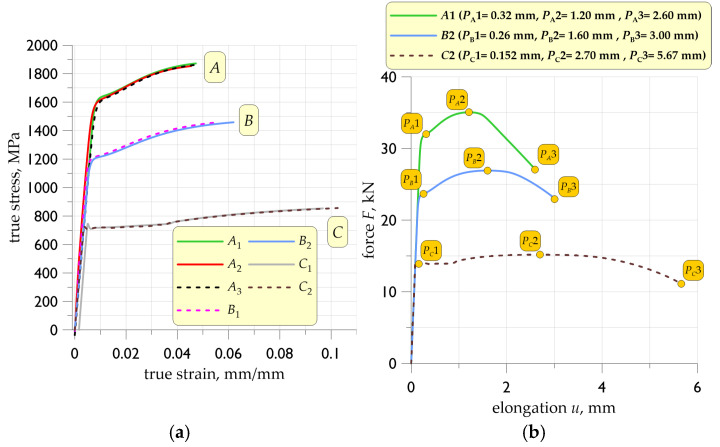
Results of tensile tests on the analysed material: (**a**) true tensile curves and (**b**) characteristic points selected for FEM analyses.

Three values of displacement applied to the specimen were assumed in the numerical calculation programme for each of the materials analysed: *P*_i_1—corresponding to the yield stress (*P*_A_1 = 0.32 mm, *P*_B_1 = 0.26 mm, *P*_C_1 = 0.15 mm), *P*_i_2—corresponding to the maximum force recorded during the laboratory test (*P*_A_2 = 1.20 mm, *P*_B_2 = 1.60 mm, *P*_C_2 = 2.70 mm) and *P*_i_3 for the critical moment (specimen rupture) (*P*_A_3 = 2.60 mm, *P*_B_3 = 3.00 mm, *P*_C_3 = 5.67 mm) (where *i* = ***A***, ***B***, ***C***—Figure 5b). The selection of several displacements as loading moments in the numerical simulation program was aimed at investigating the effect of thermal exposure on the material, its response to the applied load in particular ranges of strength characteristics.

As has been shown in many papers, the correctness of the numerical simulation results obtained is largely determined by the way the material model used in the program is described, i.e., the true stress–strain relationship. In the linear-elastic range, the material is defined by two elastic constants: Young’s modulus and Poisson’s ratio. The elastic-plastic range is described by the points from the yield point to the point of initiation of neck formation on a tensile specimen (*σ*_UTS_). At the stage of uniform elongation of the specimen during testing, the stress–strain relationship is described by the Ramberg-Osgood law of the form:

(1) $$\frac{\varepsilon }{\varepsilon_{0}}=\frac{\sigma }{\sigma_{YS}}+\alpha {\left(\frac{\sigma }{\sigma_{YS}}\right)}^{n}$$

where

*ε*_0_—strain corresponding to yield strength;

*α*—constants that depend on the material being considered;

*n*—coefficient (exponent) of material consolidation.

## 3. Results

This chapter presents the results of research conducted to assess the impact of temperature on the material of prestressing steel bars. These are the results of laboratory tests of uniaxial tensile testing with recording and analysis of acoustic emission signals, as well as the results of numerical FEM analyses.

### 3.1. Characterisation of the Material’s Strength and Ductility

Based on the results recorded during uniaxial tensile testing, the true stress–strain relationships of the steel were determined (Figure 5a). The results concerned steel in its initial state (***A***) and material subjected to heat treatment (***B*** and ***C***). The analysed steel in its initial state exhibited high strength characteristics. The tensile strength was over 1750 MPa, with a high yield strength of over 1600 MPa. With the introduction of a thermal factor during the simulation of steel behaviour in fire conditions, significant decreases in strength characteristics were observed. At a temperature of 500 °C, the tensile strength degraded by approximately 21%, and at a temperature of 700 °C, the steel had a *σ*_UTS_ value that was almost 55% lower than in its initial state. A similar trend of decreasing values with the introduction of thermal effects was observed for the yield strength. A different trend was observed in the analysis of the relative elongation of the material. At a temperature of 500 °C, the relative elongation value was similar to that of the initial state of the steel. With an increase in the annealing temperature of the material to 700 °C, a relative elongation twice as high as in the previous material variants was obtained. The true tensile curves are shown in Figure 5a, and the data are collected in Table 2. For further analysis using FEM simulations, characteristic points on the stress–strain curves were selected. The points were selected as shown in the scheme in Figure 5b.

### 3.2. Assessment of Material Damage Based on Acoustic Emission Analysis

The following section presents the interpretation of two acoustic emission parameters: Counts to Peak (CTP), which reflects the cumulative AE activity up to the moment of reaching the maximum force, and RA-value (Rise time/Amplitude), used as an indicator of the dominant cracking mechanism. These indicators make it possible to perform both qualitative and quantitative evaluation of the changes occurring in the microstructure of prestressing steel at successive stages of deformation, and they allow the distinction between quasi-brittle and ductile mechanisms depending on the thermal history of the material.

#### 3.2.1. Counts to Peak

One of the key parameters in acoustic emission analysis, widely used to assess damage initiation in metallic materials, is the number of AE counts recorded up to the point of reaching maximum load—Counts to Peak (CTP). This parameter describes the cumulative AE activity acquired from the beginning of loading until the maximum force Rm is reached. The literature [47,48,49] emphasises that CTP is sensitive both to the dominant deformation mechanism and to the microstructural condition of the material, which makes it a useful indicator of early damage processes that are not visible in conventional force–displacement curves.

In cold-drawn materials such as prestressing steel, a high Counts to Peak value is typically associated with active dislocation motion, the formation of microcracks, and local stress accumulation within the strain-hardened structure [50,51]. Conversely, a low CTP value, observed in materials after recrystallization, tempering, or high-temperature annealing, indicates a limited number of sudden AE events and the dominance of a ductile deformation mechanism controlled by dislocation slip, with the absence of residual stresses [52,53].

**Figure 6:**
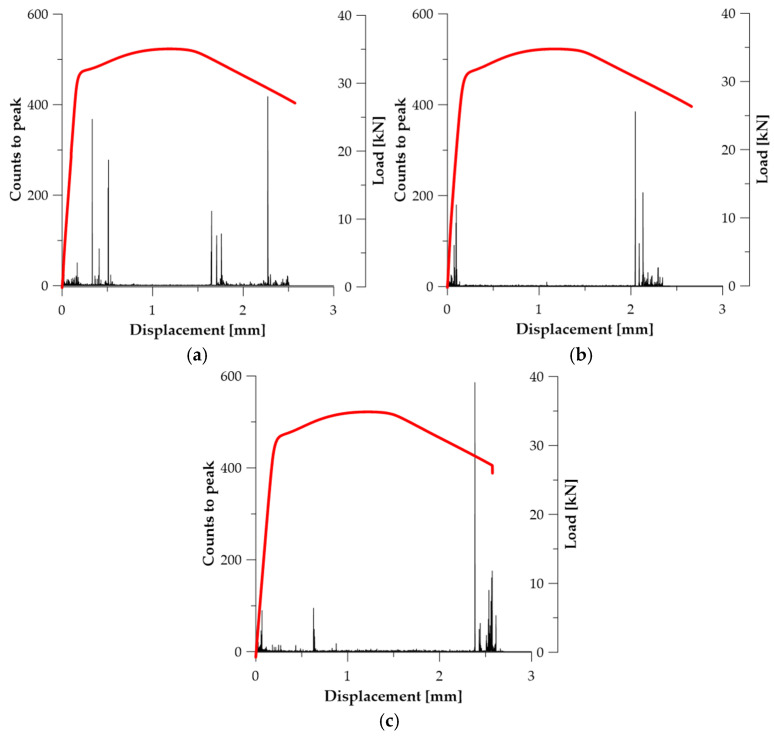
Counts to peak graphs for specimens (Load—red line): (**a**) ***A*_1_**; (**b**) ***A*_2_**; (**c**) ***A*_3_**.

In the prestressing steel specimens in the initial state (***A*_1_**, ***A*_2_**, ***A*_3_**), the Counts to Peak parameter shows a distinct intensification in the strain range exceeding the elastic limit and preceding the loss of load-carrying capacity (see Figure 6). The strongest AE bursts were recorded at

- u ≈ 2.31 mm (***A*_1_**—434 counts)—see Figure 6a;
- u ≈ 2.09 mm (***A*_2_**—384 counts)—see Figure 6b;
- u ≈ 2.39 mm (***A*_3_**—589 counts)—see Figure 6c.

This behaviour is typical of strain-hardened materials in which deformation does not develop uniformly, but transitions into a state of dynamic reorganisation of densely packed dislocations. Sudden AE bursts result from abrupt unlocking or annihilation of dislocations, their collective movement, and the nucleation of microcracks in weakened deformation bands. Therefore, the Counts to Peak parameter captures the energy associated with mechanisms responsible for the transition from diffuse to localised plasticity, which makes it a reliable marker of damage initiation in this series of tests (see Table 3).

**Figure 7:**
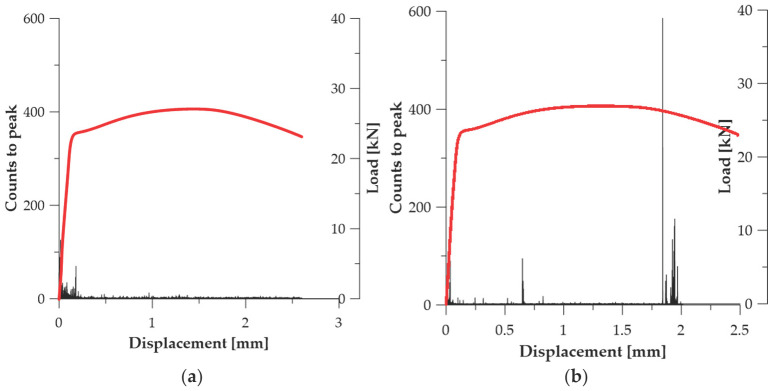
Counts to peak graphs for specimens (Load—red line): (**a**) ***B*_1_**; (**b**) ***B*_2_**.

In the steel specimens of series ***B*** (Figure 7), the intensity of the Counts to Peak parameter changed as a result of the reduction in internal stresses and the partial rearrangement of the microstructure. In specimen ***B*_1_**, the highest AE burst (131 counts) was recorded at *u* ≈ 0.164 mm, indicating a more diffuse deformation mechanism and a lower dislocation concentration in the initial plasticity range (see Figure 7a). A different behaviour was observed in specimen ***B*_2_**, where Counts to Peak reached 592 counts only at *u* ≈ 1.842 mm, i.e., in the range preceding strain localization (see Figure 7b). These data suggest that thermal relaxation of the material introduces greater deformation stability, delays the occurrence of intense AE events, and reduces the abruptness of dislocation reorganisation. In series ***B***, the Counts to Peak parameter retains its capability to register the phase leading to failure, but its evolution indicates a more ductile deformation character compared to series ***A*** (see Table 3).

**Figure 8:**
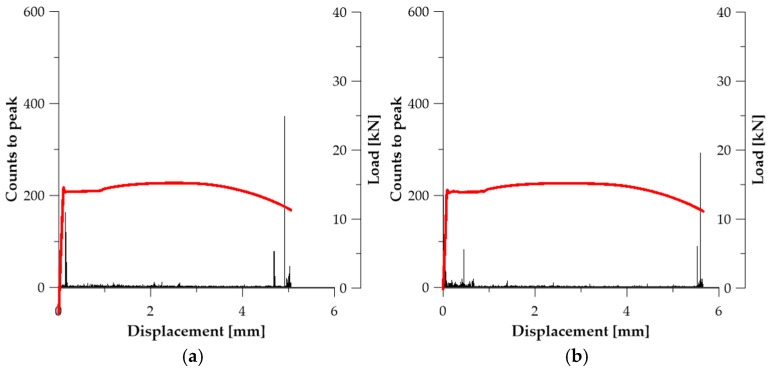
Counts to peak graphs for specimens (Load—red line): (**a**) ***C*_1_**; (**b**) ***C*_2_**.

In the specimens annealed at 700 °C (series ***C***), the behaviour of the Counts to Peak parameter corresponds to that typically observed for materials with a recrystallized microstructure, exhibiting stable and ductile deformation up to failure (Figure 8). The highest AE bursts occurred only in the final stage of material loading: at *u* ≈ 4.921 mm (***C*_1_**—CTP_max_ = 382—Figure 8a) and *u* ≈ 5.525 mm (***C*_2_**—CTP_max_ = 300—Figure 8b). This indicates that AE activity remained low for most of the test duration, and only necking formation and final material separation generated intense emission. This mechanism is characteristic of materials with high plastic deformability, in which AE activity arises not from dislocation motion, but from crack-surface friction and macroscopic crack opening (see Table 3). Counts to Peak is therefore the most effective parameter for assessing the onset of failure in series ***C***.

#### 3.2.2. RA-Value

The RA-value parameter (Rise time/Amplitude) is an indicator used to identify the material damage mechanism. It is widely accepted in the literature that low RA-values correspond to sudden phenomena such as brittle cracking, which generate signals with high amplitude and short rise time. In contrast, high RA-values are characteristic of ductile mechanisms, in which dislocation slip, crack-surface friction, and gradual material separation dominate—producing longer signals with lower instantaneous energy [54,55].

For strain-hardened materials such as cold-drawn prestressing steel, RA-values remain relatively high in the initial stage of deformation (microplasticity). However, near the moment of crack initiation, they drop sharply, which is a sign of a transition to a brittle or brittle–plastic failure mechanism [56].

**Figure 9:**
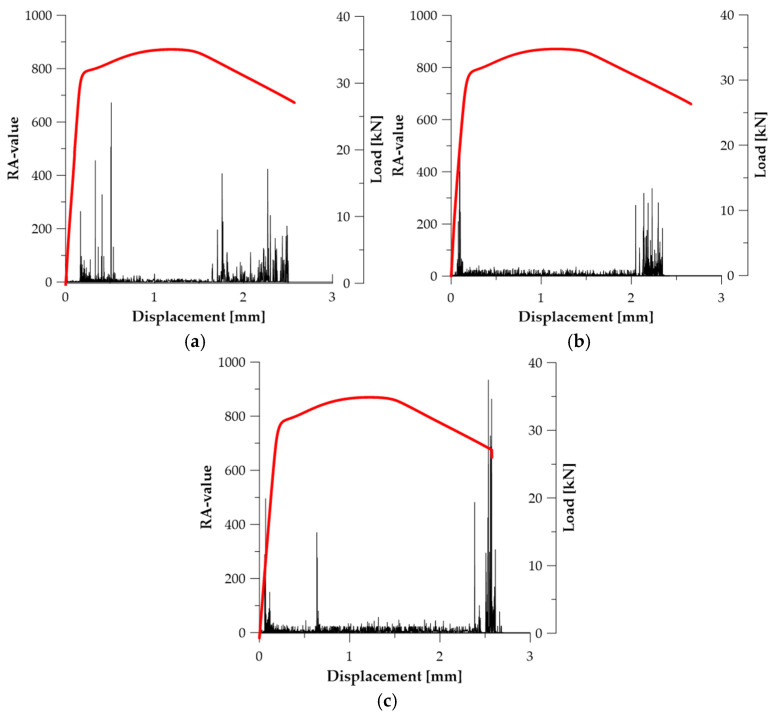
RA-value graphs for specimens (Load—red line): (**a**) ***A*_1_**; (**b**) ***A*_2_**; (**c**) ***A*_3_**.

In the case of series, ***A*** (see Figure 9), RA-value reached its maxima significantly earlier than Counts to Peak, which indicates that this parameter primarily captured the earliest transition from microplasticity to active dislocation deformation, before the onset of progressive strain localization in the material (Figure 9a). For specimen ***A*_2_**, the maximum RA-value RA_max_ = 520.0 occurred at *u* ≈ 0.052 mm (see Figure 9b), while in specimen ***A*_3_**, RA_max_ = 933.93 was recorded at *u* ≈ 2.691 mm (see Figure 9c). The RA-value characteristics for series ***A*** reveal the dominance of signals with short rise time and relatively high amplitude, typical of sudden dislocation avalanches, without the involvement of crack-surface separation mechanisms characteristic of ductile fracture (see Table 3).

**Figure 10:**
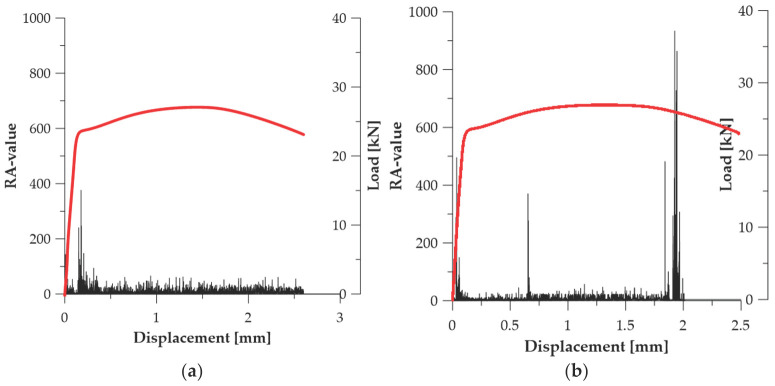
RA-value graphs for specimens (Load—red line): (**a**) ***B*_1_**; (**b**) ***B*_2_**.

In series ***B*** (Figure 10), the RA-value parameter exhibits a distinctly diversified behaviour resulting from partial homogenization of internal stresses and the transition of the material toward a more ductile deformation mode. In specimen ***B*_1_**, RA_max_ = 376.51 was recorded at *u* ≈ 0.177 mm (see Figure 10a), while in specimen ***B*_2_** the value RA_max_ = 933.93 occurred at *u* ≈ 1.925 mm (see Figure 10b), i.e., within the region of localised deformation. This indicates that in this series, the RA-value captures both early dislocations slip activity and later, slower frictional mechanisms associated with material separation in the necking zone (see Table 3).

**Figure 11:**
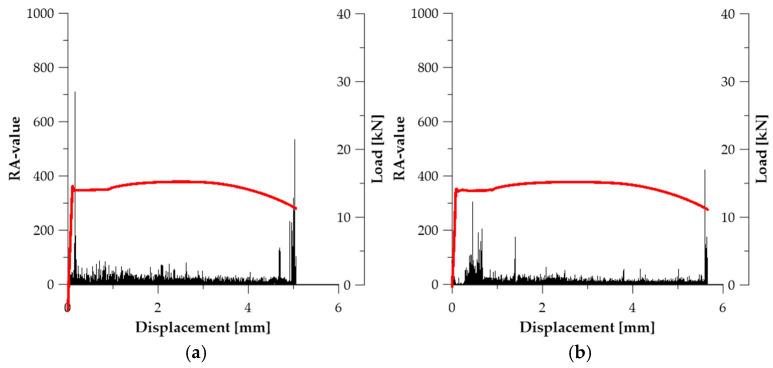
RA-value graphs for specimens (Load—red line): (**a**) ***C*_1_**; (**b**) ***C*_2_**.

In series ***C*** (Figure 11), the RA-value remained at a low level for most of the deformation process, which indicates the predominance of slow, non-violent deformation mechanisms. In specimen ***C*_1_**, RA_max_ = 709.83 was recorded at *u* ≈ 0.165 mm (see Figure 11a), whereas in specimen ***C*_2_**, RA_max_ = 423.05 appeared only at *u* ≈ 5.598 mm (see Figure 11b), which coincides with the moment of failure. This result suggests that in series ***C***, the RA-value reflects the contribution of frictional and separation processes characteristic of ductile fracture, preceded by a long stage of stable deformation (see Table 3). Therefore, in this series, the RA-value parameter has the highest diagnostic value among all analysed material states.

**Table 3:** Summary of AE characteristics for the three material states.

Material State	Dominant AE Mechanism	Counts to Peak Behaviour	RA-Value Behaviour	Interpretation
***A* (20 °C)**	sudden dislocation avalanches, early microcracking	sharp peaks early in plastic range	high at onset, decreasing near fracture	quasi-brittle response
***B* (500 °C)**	mixed dislocation slip + ductile friction	delayed peaks, more diffuse	moderate, rising toward necking	transitional behaviour
***C* (700 °C)**	ductile separation, frictional mechanisms	peaks only near failure	low throughout, increasing slightly	fully ductile behaviour

### 3.3. Numerical Simulations

**Figure 12:**
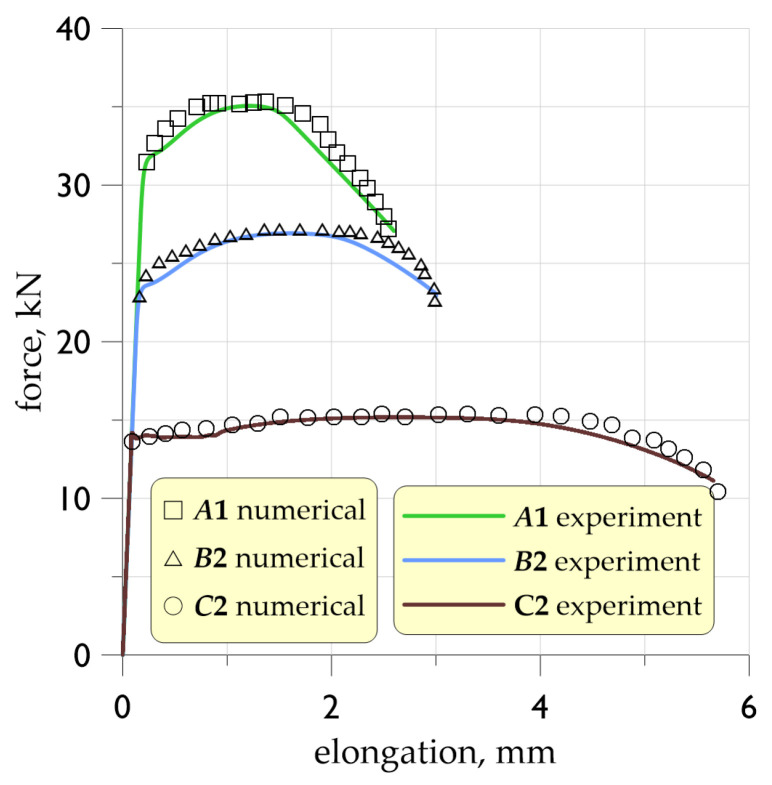
Numerical calculations details: preparation of the material model for use in numerical simulations: convergence of force–displacement relationships.

In FEM calculations for materials with significant levels of plasticity, the indicated form of material model definition is not sufficient. It is necessary to define the critical stress and strain levels characteristic of the material. The prepared true stress–strain relationship was subjected to a calibration procedure involving multiple iterative calculations. This method took into account the distribution in the analysed geometry: the stress triaxiality factor *η* and the Lode parameter. The target point of the calibration procedure was to achieve convergence of the load curves as a function of displacement from two sources: that determined in laboratory tests and that determined as a result of FEM numerical simulations. It was assumed that a convergence of both curves at the level of 5% would allow the developed numerical model and the obtained results to be considered reliable. The criterion for the calibration procedure of the *σ*-*ε* relation in this paper is the agreement of the experimental and numerical load curves (Figure 12). The calibration procedure was carried out for each variant of thermal exposure on the material.

As a result of the numerical simulations, the individual quantities characterising the material of the specimens under load were determined: the Mises effective stress, the component of the stress tensor acting in the direction of the applied load (*σ*_22_), the effective plastic strain *ε*_pl_eff_ and the stress triaxiality factor *η*, which is defined by the relation [57]:

(2) $$\eta =\frac{\sigma_{m}}{\sigma_{eff}}=\frac{\frac{1}{3}\left(\sigma_{11}+\sigma_{22}+\sigma_{33}\right)}{{\left\{\frac{1}{2}{[\left(\sigma_{11}-\sigma_{22}\right)}^{2}+{\left(\sigma_{11}-\sigma_{33}\right)}^{2}+{\left(\sigma_{22}-\sigma_{33}\right)}^{2}]\right\}}^{\frac{1}{2}}}$$

where:

*σ*_m_—medium stress;
*σ*_eff_—effective von Mises stress;
*σ*_11_, *σ*_22_, *σ*_33_—components of the stress tensor.

**Figure 13:**
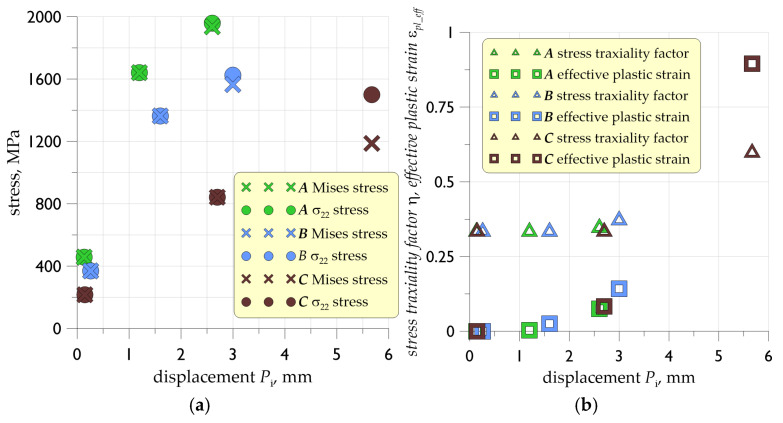
Numerically determined maximum values of the individual quantities characterising the state of the material: (**a**) Mises and *σ*_22_ stress, (**b**) stress triaxiality factor and effective plastic strain.

The highest stress levels were observed in the initial material, without the influence of thermal effects. Across the entire load range under analysis, the *σ*_22_ stresses and effective stresses exhibited comparable values (Figure 13a). At the maximum displacement for the maximum force, the stresses were approximately 1600 MPa, while at the point of rupture they were in the region of 2000 MPa (see Figure 13a). The introduction of a temperature interaction of 500 °C (material ***B***) resulted in a reduction in the numerically determined stress levels. The stress reduction at the point of maximum force (*P*_B_2) and at the critical moment (*P*_B_3) was approximately 17%. In the yield point region, the numerically determined stresses for material ***A*** were approximately 19% higher than in material ***B***. The lowest stress levels were recorded for the material annealed at 700 °C. At the *P*_c_2 point, the *σ*_22_ and effective stresses were reduced by approximately 50% relative to material ***A***, while approximately 38% relative to material annealed at 500 °C (***B***). The reduction in stress levels at the critical moment was less pronounced, with a maximum of approximately 23% relative to the original material (Figure 13a).

The stress triaxiality factor *η* for materials ***A*** and ***B*** was in the range of 0.33 over the entire load range tested. The occurrence of relatively high levels of plastic deformation for material ***C*** at critical rupture (for *P*_c_3 *ε*_pl_eff_= 90%) resulted in an approximate value of the *η* coefficient of 0.6. In comparison, materials ***A*** and ***B*** exhibited relatively low values of plastic deformation over the entire load range, reaching a maximum of 0.14 for point *P*_B_3 (Figure 13b).

## 4. Discussion

The acoustic emission response and FEM-derived mechanical fields jointly indicate a clear, temperature-dependent evolution of deformation and failure mechanisms in prestressing steel. In the initial cold-drawn condition (series ***A***), the material exhibits behaviour characteristic of a strain-hardened microstructure with high internal stresses. The very early RA-value maxima (e.g., RA_max_ = 520 at u ≈ 0.052 mm for ***A*_2_**) reflect abrupt dislocation avalanches, whereas the highest Counts to Peak values recorded later (u ≈ 2.31–2.39 mm) correspond to the development of strain localisation and the initiation of microcracks. FEM results support this interpretation: *σ*_22_ reaches 1850–1950 MPa at relatively small axial strains (*ε*_22_ ≈ 0.04–0.08), followed by a rapid loss of load-bearing capacity. Together, these findings confirm a quasi-brittle failure mode driven by instability within the strain-hardened microstructure.

After annealing at 500 °C (series ***B***), the AE and FEM results indicate a transitional deformation mechanism. Partial stress relaxation reduces the energy of early AE events (e.g., RA_max_ ≈ 376 at u ≈ 0.18 mm for ***B*_1_**), and pronounced AE activity appears only near the development of strain localisation (CTP_max_ ≈ 592 at u ≈ 1.84 mm for ***B*_2_**). FEM analysis shows lower peak stresses (≈1400–1600 MPa) and larger axial strains (*ε*_22_ ≈ 0.10–0.15), demonstrating that plastic flow becomes more stable and that damage initiation is delayed relative to series ***A***. Failure in this state is governed by progressive localisation and frictional separation rather than sudden, brittle microcracking.

In the specimens annealed at 700 °C (series ***C***), the combined AE–FEM response is consistent with a fully ductile failure mechanism. Recrystallization removes the strain-hardening effect, leading to minimal AE activity until the final loading stage. RA-value maxima occur late (e.g., RA_max_ = 423 at u ≈ 5.598 mm for ***C*_2_**), and Counts to Peak increases only immediately before rupture (CTP_max_ ≈ 300–382 at u ≈ 4.9–5.5 mm). Correspondingly, FEM simulations show low stresses (*σ*_22_ ≈ 800–1200 MPa) and very large axial strains (*ε*_22_ ≈ 0.40–0.90), confirming that deformation proceeds by uniform plastic flow with delayed localisation.

Comparing the three thermal states demonstrates a systematic shift from quasi-brittle behaviour (***A***) toward transitional (***B***) and fully ductile (***C***) mechanisms. This trend is consistently captured by both AE parameters:

- RA-value identifies whether deformation is dominated by abrupt or gradual processes,
- Counts to Peak marks the onset of strain localisation,
- *ε*_22_ and *σ*_eff_ describe the corresponding mechanical state.

On this basis, a multi-criteria indicator for identifying the onset of load-bearing capacity loss was formulated. Failure initiation occurs when

- Counts to Peak reaches a critical level characteristic of a given material condition, evaluated in the damage initiation window (near the onset of strain localisation), with a characteristic change in the RA-value:
  ○ CTP ≈ 350–600 and RA ≈ 100–300, corresponding to an impulsive AE response with pronounced, short-duration peaks and no sustained elevated RA level in the damage initiation stage, which is typical of a strain-hardened, quasi-brittle deformation mechanism (***A***);
  ○ CTP ≈ 250–600 and RA ≈ 200–500, with the maximum RA correlated with the strain localisation area, indicating a transitional deformation mechanism with a mixed contribution of dislocation slip and frictional–ductile processes (***B***);
  ○ CTP ≈ 300–380 and RA ≥ 300–450, with low characteristic RA values during most of the loading process and a pronounced increase in both parameters immediately before fracture, which is characteristic of a fully ductile failure mechanism governed by crack-surface separation and friction (***C***).
- axial strain *ε*_22_ exceeds
  ○ 0.04–0.08 (***A***);
  ○ 0.10–0.15 (***B***);
  ○ 0.40–0.50 (***C***).
- von Mises stress *σ*_eff_ reaches its maximum and begins to decline:
  ○ 1850–1950 MPa (***A***);
  ○ 1400–1600 MPa (***B***);
  ○ 800–1500 MPa (***C***).

To separate pre-damage behaviour from true damage initiation, an additional elastic–microplastic criterion was introduced. The material is considered to remain in the safe, pre-damage state when AE activity is diffuse and low-energy (Counts to Peak ≲ 10–20), RA-value does not show a consistent trend toward cracking, *ε*_22_ stays below the thresholds for damage initiation, and *σ*_eff_ increases monotonically without a post-peak reduction.

Overall, the integration of AE data with FEM-derived stress and strain fields provides a coherent and physically grounded framework for interpreting temperature-induced degradation mechanisms. The proposed criteria—validated across the three thermal conditions, enable precise identification of the transition from stable plasticity to damage initiation and offer a robust basis for assessing the residual load-bearing capacity of prestressing steel after fire exposure.

## 5. Conclusions

This study investigated the mechanical response, microstructural evolution and acoustic-emission behaviour of cold-drawn prestressing steel after exposure to high temperatures. Based on the combined experimental and numerical analyses, the main scientific contributions of this work are as follows:

- Identification of microstructural and mechanical degradation trends in prestressing steel after exposure to 500 °C and 700 °C, including strength reduction, ductility changes, and temperature-dependent microstructural transformations;
- Demonstration of distinct AE signatures associated with quasi-brittle (20 °C), transitional (500 °C), and ductile (700 °C) failure modes, reflecting the underlying deformation and damage mechanisms;
- First-time correlation of AE parameters with FEM-derived strain and stress fields, enabling objective identification of the onset of damage and strain localization in fire-exposed prestressing steel;
- Development of a multi-criteria AE–FEM threshold, integrating Counts to Peak, RA-value, *ε*_22_ and *σ*_eff_, for assessing the onset of load-bearing capacity loss;
- Validation of AE as a practical non-destructive diagnostic tool for post-fire evaluation of prestressing tendons, offering potential for structural safety assessment after thermal events.

The main limitations of this study arise from the relatively small number of specimens and the restriction to uniaxial tensile loading, which does not reproduce the multi-axial stress states present in real prestressing tendons. In addition, only two AE parameters were analysed, and event localisation was not performed, which limits the spatial resolution of damage detection. Future work should therefore incorporate multi-sensor AE arrays, thermomechanical coupling in FEM analyses, and validation on full-scale prestressing cables subjected to realistic fire scenarios.

## Figures and Tables

**Table 1:** Maximum values of signals for individual quantities recorded during uniaxial tensile tests.

Material	*F*_max_, kN	*u*_max_, mm
***A*** **_1_**	35.05	2.59
***A*** **_2_**	34.77	2.66
***A*** **_3_**	34.87	2.57
***B*** **_1_**	27.07	2.60
***B*** **_2_**	26.91	3.00
***C*** **_1_**	15.21	5.07
***C*** **_2_**	15.17	5.66

**Table 2:** Strength and plasticity characteristics of the material.

Material	*σ*_YS_, MPa	*σ*_UTS_, MPa	*E*, GPa	*EL*, %
***A*** **_1_**	1640.12	1869.86	207	10.29
***A*** **_2_**	1615.5	1853.06	202	10.64
***A*** **_3_**	1604	1863.98	199	10.30
***B*** **_1_**	1215.5	1458.39	204	10.40
***B*** **_2_**	1210	1457.16	203	11.90
***C*** **_1_**	710.22/ 745.87	853.61	206	20.30
***C*** **_2_**	706.84/ 725.88	856.24	207	22.63

where *σ*_YS_—yield strength; *σ*_UTS_—ultimate tensile strength; *E*—Young’s modulus; *EL*—total elongation.

## Data Availability

The data presented in this study are available on request from the corresponding author, the data are not publicly available due to privacy restrictions.

## Abbreviations

The following abbreviations are used in this manuscript:

*σ*_YS_	yield strength
*σ*_UTS_	ultimate tensile strength
*E*	Young’s modulus
*EL*	total elongation
ε_22_	axial strain component in the loading direction
σ_22_	axial stress component in the loading direction
*σ*_eff_	von Mises effective stress
*ε*_pl_eff_	effective plastic strain
*η*	stress triaxiality factor
*σ*_m_	medium stress
CTP (Counts to Peak)	cumulative number of AE counts
RA-value (Rise time/Amplitude)	AE parameter indicating fracture mechanism
***A***, ***B***, ***C***	material variants
*P*_i_1, *P*_i_2, *P*_i_3	numerical loading points for variant i (yield point, maximum force, failure)
FEM	Finite Element Method
