# Correction: Adamczak-Bugno et al. Evaluation of the Effect of Temperature (20–700 °C) on the Properties of Prestressing Steel Using AE Signals and FEM Analysis. *Materials* 2026, *19*, 23

**DOI:** 10.3390/ma19040739

**Published:** 2026-02-14

**Authors:** Anna Adamczak-Bugno, Sebastian Michał Lipiec, Jakub Adamczak

**Affiliations:** 1 Faculty of Civil Engineering and Architecture, Kielce University of Technology, Av. 1000-An. of Polish State 7, 25-314 Kielce, Poland; jakubadamczak123@gmail.com; 2 Faculty of Mechatronics and Mechanical Engineering, Kielce University of Technology, Av. 1000-An. of Polish State 7, 25-314 Kielce, Poland; slipiec@tu.kielce.pl

There were some errors in the original publication [1]. Some texts need to be updated in Sections 3.2.1 and 4.

In Section 3.2.1, paragraph 3, the sentence

- u ≈ 0.44 mm (***A*_1_**—384 counts)—see Figure 6a;
- u ≈ 1.09 mm (***A*_2_**—334 counts)—see Figure 6b;
- u ≈ 1.59 mm (***A*_3_**—389 counts)—see Figure 6c.

should be updated to the following version:

- u ≈ 2.31 mm (***A*_1_**—434 counts)—see Figure 6a;
- u ≈ 2.09 mm (***A*_2_**—384 counts)—see Figure 6b;
- u ≈ 2.39 mm (***A*_3_**—589 counts)—see Figure 6c.

In Section 3.2.1, paragraph 5, the sentence “In specimen ***B*_1_**, the highest AE burst (331 counts) was recorded at *u* ≈ 0.164 mm, indicating a more diffuse deformation mechanism and a lower dislocation concentration in the initial plasticity range (see Figure 7a).” should be updated to the following version:

“In specimen ***B*_1_**, the highest AE burst (131 counts) was recorded at *u* ≈ 0.164 mm, indicating a more diffuse deformation mechanism and a lower dislocation concentration in the initial plasticity range (see Figure 7a).”

In Section 4, paragraph 1, the sentence “The very early RA-value maxima (e.g., RA_max_ = 520 at u ≈ 0.052 mm for ***A*_2_**) reflect abrupt dislocation avalanches, whereas the highest Counts to Peak values recorded later (u ≈ 0.44–1.59 mm) correspond to the development of strain localisation and the initiation of microcracks.” should be updated to the following version:

“The very early RA-value maxima (e.g., RA_max_ = 520 at u ≈ 0.052 mm for ***A*_2_**) reflect abrupt dislocation avalanches, whereas the highest Counts to Peak values recorded later (u ≈ 2.31–2.39 mm) correspond to the development of strain localisation and the initiation of microcracks.”

In Section 4, paragraph 5, the sentence

- Counts to Peak ≥ 530–600 and characteristic RA-value change:
  ∘ RA ≤ 50–150 (***A***);
  ∘ RA ≈ 100–300 (***B***);
  ∘ RA ≥ 300–450 (***C***).

should be updated to the following version:

- Counts to Peak reaches a critical level characteristic of a given material condition, evaluated in the damage initiation window (near the onset of strain localisation), with a characteristic change in the RA-value:
  ∘ CTP ≈ 350–600 and RA ≈ 100–300, corresponding to an impulsive AE response with pronounced, short-duration peaks and no sustained elevated RA level in the damage initiation stage, which is typical of a strain-hardened, quasi-brittle deformation mechanism (***A***);
  ∘ CTP ≈ 250–600 and RA ≈ 200–500, with the maximum RA correlated with the strain localisation area, indicating a transitional deformation mechanism with a mixed contribution of dislocation slip and frictional–ductile processes (***B***);
  ∘ CTP ≈ 300–380 and RA ≥ 300–450, with low characteristic RA values during most of the loading process and a pronounced increase in both parameters immediately before fracture, which is characteristic of a fully ductile failure mechanism governed by crack-surface separation and friction (***C***).

In Section 4, paragraph 5, the sentence “1200–1500 MPa (***C***).” should be updated to “800–1500 MPa (***C***).”

The authors state that the scientific conclusions are unaffected. This correction was approved by the Academic Editor. The original publication has also been updated.
